# Mismatch repair deficiencies transforming stem cells into cancer stem cells and therapeutic implications

**DOI:** 10.1186/1476-4598-6-26

**Published:** 2007-04-02

**Authors:** Minal Vaish

**Affiliations:** 1 Department of Biochemistry, University of Lucknow – 226007, U.P., India

## Abstract

For the exceptional self-renewal capacity, regulated cell proliferation and differential potential to a wide variety of cell types, the stem cells must maintain the intact genome. The cells under continuous exogenous and endogenous genotoxic stress accumulate DNA errors, drive proliferative expansion and transform into cancer stem cells with a heterogeneous population of tumor cells. These cells are a common phenomenon for the hematological malignancies and solid tumors. In response to DNA damage, the complex cellular mechanisms including cell cycle arrest, transcription induction and DNA repair are activated. The cells when exposed to cytotoxic agents, the apoptosis lead to cell death. However, the absence of repair machinery makes the cells resistant to tumor sensitizing agents and result in malignant transformation. Mismatch repair gene defects are recently identified in hematopoietic malignancies, leukemia and lymphoma cell lines. This review emphasizes the importance of MMR systems in maintaining the stem cell functioning and its therapeutic implications in the eradication of cancer stem cells and differentiated tumor cells as well. The understanding of the biological functions of mismatch repair in the stem cells and its malignant counterparts could help in developing an effective novel therapies leaving residual non-tumorigenic population of cells resulting in potential cancer cures.

## Background

Gurdon first introduced the concept of stemness state of the cells while successfully achieving the generation of all cell lineages of a living tadpole after transferring the nuclei from intestinal epithelial cells of feeding Xenopus tadpoles to activated enucleated eggs [1,2]. These types of cells were later on characterized as indispensable entities, identified in multicellular organisms. The stem cell phenotype is contributed by the unique features that include (1) self-renewal which means that after cell division, one of the daughter cells must have the same genetic material as that of the parent cell, (2) differentiation i.e. capability to differentiate into multiple lineages and (3) extensive proliferation. The adult stem cells are identified in variety of tissues and organs in humans including – bone marrow, brain, skin/hair follicles, heart, lung, intestine, liver, pancreas, mammary glands, ovaries, prostrate, and testis [3].

**Figure 1:**
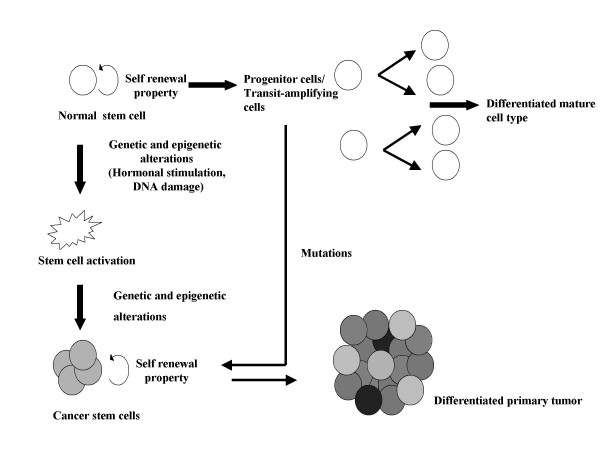
Development of cancer stem cells from the normal stem cells and progenitor cells. Accumulation of DNA errors in normal stem cells or progenitor cells are activated to generate a cancer stem cells (CSCs) that further generate a primary tumor constituting CSCs and other tumor cells.

The multipotency of stem cells to maintain tissue homeostasis and its differentiation into mature cell types is under a tightly controlled system and is associated with restricted expression profile. The expression of transport proteins – ABC (ATP-binding cassette) transporter proteins and multidrug resistant proteins that protect cells against toxins and are associated with the efflux of xenobiotic toxins, low rate of cell division and active DNA repair are the innate properties of normal development of stem cells. Besides hormonal stimulation, DNA damage is one of the key factors for stem cell activation. DNA accumulates errors either from environmental factors, which could be the exposure to radiation, chemicals or drugs, viruses and bacteria or DNA replication errors [4]. This would result in complex cellular responses that include loss of cell cycle regulation, transcription induction and DNA repair mechanisms for maintaining genomic and chromosomal stability. But in absence of efficient DNA repair machinery, the stem cells accumulate harmful mutations and become resistant to apoptosis resulting in the loss of genome or instability, which ultimately lead to malignant transformation of stem cells [5]. Table 1 features the stem cell activation types, which transforms them into different cancer types [4,6]. Each stem cell activation type is exemplified with a single cancer type in the Table 1, however many could be associated. The progenitor cells also known as transit – amplifying cells arise from normal stem cells have replicative ability but not self-renewal capacity. Mutations in these progenitor cells help in regaining self-renewal property and become cancerous (Fig 1).

**Table 1:** Stem cell activation types transforming normal stem cells into cancerous stem cells

***Stem cell activation type***	***Target tissue/organ***	***Cancer type***
Naturally activated stem cell: Inactivation of RB1 gene	Retinoblasts	Retinoblastoma
Loss of tumor suppressor genes (p53)	Breast	Breast
Expression of oncogenes (ras, myc)	Brain	Brain
Hormonal stimulation: estrogen	Breast	Breast
Inflammation: Crohn disease, inflammatory bowl disease, result in activated cell growth	Colon	Colon
Viral infection: Hepatitis B and C cause inflammation and extensive cirrhosis	Liver	Liver
Exposure to irritants like tobacco, asbestos cause inflammation	Lung	Lung
Bacterial infection: Helicobacter pylori and metal dust exposure cause inflammation	Stomach	Stomach
Loss of miRNA genes (miR15 and miR16) which act as tumor suppressors	Bone marrow	Chronic lymphoid leukemia
Enforced expression of miR17-92 cluster which acts as oncogenes	Bone marrow	B cell lymphoma
DNA methylation at 5-position at cytosine residue within CpGs by Dnmt1 (maintenance methyltranferase), Dnmt3a, 3b (initiate de novo methylation), Dnmt2	Colon	Colorectal
Methylation dependent repression of transcription by binding of methyl CpG binding proteins- MECP2, MBD1-4, Kaiso to DNA	Colon	Colorectal
Histone methylation by H3K4 Mtases, H3K9 Mtases, Suv39h1/Suv39h2, G9a, Eu-H Matse1, ESET/SETDB1	Prostate	Prostate
Histone acetylation via histone acetyltransferases (HAT) include Gcn5 family proteins, MYST protein, p300/CBP, TAF250, ACTR/SRC1 nuclear receptor cofactors mediate transcriptional activation	Breast	Breast
Histone deacetylation silences gene expression via HDAC I family, HDAC II family, Sirtuin family (Sir2)	Bone marrow	Acute myeloid leukemia
Blockage of DNA accessibility to transcription factors by polycomb group proteins which include Polycomb repressive complexes – PRC1 contains Cbx, Mph, Ring, Bmi-1, Mel18 and PRC2 contains Ezh2, Suz12 and Eed	Bone marrow	B and T cell lymphoma
Alteration in chromatin accessibility to proteins and restriction endonucleases by the disruption of association of histones with DNA using the energy by ATP hydrolysis via ATP dependent remodeling complexes (SWI2/SNF2 protein, ISWI enzymes, Mi-2/NuRD proteins	Bone marrow	Acute myeloid leukemia

Cancer stem cells (CSCs) were first identified in 1990s in hematological malignancies, mainly acute myelogenous leukemia (AML) and also in other subtypes like AML M0, M1, M2, M4 and M5 and chronic myeloid leukemia (CML), acute lymphoblastic leukemia (ALL) and multiple myeloma [7]. CSCs are also known in solid tumors like breast, brain, lung, prostrate, testis, ovary, stomach, colon, skin, liver, pancreas [8-10]. These tumor cells have a self-renewal property but undergo aberrant differentiation and constitute the heterogeneous population of tumor cells [11].

In the hematopoietic and solid tumors side population (SP) containing CSCs constitute the fraction of total population of cells that shows that CSCs is a common phenomenon for all the malignancies. SP can be identified by the efflux of fluorescent dyes by ABC transporter proteins which are the major multidrug resistant genes. In the absence of DNA repair capacity, the drug resistant pluoripotent cells accumulate mutations and show increased cell survival [12].

### DNA repair

To maintain the genomic integrity and normal functioning of stem cells, several DNA repair pathways come into interplay and provide the robust defense to the cell. Absence of these repair processes have a great impact on the diminution of stem cells and increased chance for stem cell differentiation and malignant transformation due to altered gene expression.

#### (a) O6- alkylguanine DNA alkyl transferase (AGT) repair

AGT repair is a DNA repair protein encoded by MGMT (O6-methylguanine DNA methyltransferase) removes DNA adducts from O6 methylguanine. The failure in the repair system results in G to A transition and DNA strand break observed in hematopoietic tumors, breast, ovarian and retinoblastomas [13,14].

#### (b) Nucleotide Excision Repair (NER)

In NER, the damaged DNA bases are recognized by XPC (xeroderma pigmentosum complementation group C) and RAD23B whereas XPA, RPA (replication protein A), RNA polymerase II transcription factor 11A and XPG excise the fragment of 27–30 nucleotides surrounding the damaged bases. The gap is further restored by DNA polymerases and ligases and replaces it with the correct sequence. Defective NER leads to xeroderma pigmentosum (skin cancer) [15].

#### (c) Base Excision Repair (BER)

It comprises of short and long patch pathways where DNA glycosylases generate apurinic/apyrimidinic (AP) followed by its 5' excision by AP endonucleases (APE-1) and remove damaged bases. Double-stranded breaks have the potential to be the most disruptive form of DNA damage [16,17].

#### (d) Non-Homologous DNA End-Joining (NHEJ) repair

Double-stranded breaks have the potential to be the most disruptive form of DNA damage. The DNA double strand breaks (DSBs) produced by free radicals generated during oxidative respiration, ionizing radiation, DNA replication, malfunction of recombination activating gene complex during V(D)J (variable region, diversity region-junction region) recombination in T and B lymphocytes are brought in closed proximity by Ku70-Ku80 heterodimer and kinase activity by DNA dependent protein kinase catalytic subunit (DNA-PKcs) of DNA-PK complex followed by its ligation by XRCC4-DNA ligase IV, SCID (Severe Combined Immunodeficiency) is associated with defective NHEJ repair system [15].

#### (e) Homologous Recombination (HR) repair

The DSBs are repaired by misalignment, deletions, and rearrangement. ATM, a PI-3 Kinase binds DNA and phosphorylates multiple proteins. BRCA1 activated by ATM (ataxia telangiectasia) facilitates BRCA2 and RAD51 binding of the overhang followed by the attraction of RAD52/RAD54 with the help of BLM/WRN proteins. The homologous recombination is facilitated by large protein complexes at the DSBs. High incidence of leukemia, breast-ovarian, Werner's and Bloom's syndrome with severe premature aging and cancer is seen in defective HR repair system [18,19].

#### (f) Mismatch Repair (MMR)

It is a genome surveillance system in bacteria, yeast and mammals by maintaining genomic integrity. The MMR pathway is the form of DNA repair responsible for the elimination of specific mismatched and/or unmatched bases and insertion-deletion loops (IDLs) where DNA synthesis is liable to errors, either as a part of DNA replication before cell division, or as part of DNA repair (unscheduled DNA synthesis) [20]. Although DNA polymerases have the ability to identify and correct their own errors, some mistakes are not identified.

Mismatch in eukaryotic DNA is recognized by two heterodimeric complexes of Mut S related proteins – MSH2/GTBP (Mut Sα) and MSH2/MSH3 (Mut Sβ) [21]. Mut Sα binds to both base – base mismatches and small ID (insertion – deletion) heterologies whereas Mut Sβ plays major role in repair of larger ID mispairs [22]. Presence of ATP markedly decreases the affinity of Mut Sα for an oligonucleotide heteroduplex, an effect also observed with bacterial Mut S. Hydrolysis of ATP facilitates protein – protein interactions and or sliding along with DNA [23]. Recognition of mispair is followed by binding of Mut Sα (MSH2/MSH3) with Mut L related proteins [MLH1/hPMS2 (PMS1 in yeast)] and converts into high molecular weight structure. It also increases the efficiency of Mut S proteins to recognize the mismatch. MLH1/MLH3 (PMS2 in humans) also forms the complex with Mut Sβ and help in repair of ID mispairs [24].

Proliferating cell nuclear antigen (PCNA) has been identified as DNA polymerase processivity factor and has a role in repair at or prior to excision steps as a strand discriminating factor [25]. Bi – directional threading of DNA through hMut S heterodimer is continued till the arrest of forward movement of replication fork that occurs via an interaction with PCNA in the polymerase complex. Recently another eukaryotic mismatch repair endonuclease has been identified whose amino terminal domain is involved in binding to fully methylated DNA and carboxyl terminal region is involved in catalysis and complex formation with Mut L homologue MLH1. This protein is named as MED1 (methyl – CpG binding endonuclease 1). The properties of MED1 felicitate it to be a functional homologue of MutH [26].

DNA exonuclease and helicase unwind, nick and degrade the error – containing strand. After dissociation of MMR complex, PCNA, which still binds at the end of error containing primer strand, recruits replication complex and thus re-initiation starts with the help of DNA polymerase.

The mutations characterized by point mutations, insertions or deletions in the length of DNA microsatellite repeat sequences throughout the genome resulting in microsatellite instability (MSI) is a hallmark of defective DNA MMR system which result in malignant transformation [27].

The maintenance of MMR system decreases the error rates by 100 to 1000 fold during DNA replication. Since it has tremendous importance in the carcinogenesis, this review presents a discussion on the role of DNA mismatch repair system as a therapeutic potential in eliminating the cancer stem cells.

### MMR deficiency and stem cell defects

The maintenance and longevity of stem cell phenotype is characterized by the presence of efficient MMR system, which result in accurate DNA replication, restore normal DNA after its damage and remove replication defects at microsatellite sequences by repairing the DNA polymerase slippage products.

The MMR deficiencies because of the mutations in wild type MMR allele with Msh2^-/- ^and Mlh2^-/- ^phenotype, mice develop stem cell-derived-lymphoid malignancies and all lymphoid tumors show MSI [28]. The serial bone marrow transplantation assay was done to compare Msh2^-/- ^primary murine hematopoietic cells with wild type into lethally irradiated mice with temozolomide, a methylating agent. MMR deficiency as evident by MSI was observed in early progenitor colony forming unit (CFU) and Sca^+^Kit^+^Lin^-^ derived clones, which explain the alteration in growth and survival of hematopoietic stem cells and its long-term repopulation capacity because of accumulation of genomic instability [5]. In another study, colonogenic survival was assayed in Msh2^-/-^, Msh2^+/+^, Msh2^-/+ ^mouse embryonic stem cells following prolonged low level radiation treatment. The cells deficient in active MMR system survive promutagenic genomic insults by alkylating and oxidating agents that contribute to neoplastic transformation as in hereditary non-polyposis colorectal cancer (HNPCC) [29]. The six cases of childhood leukemia are reported where patients are homozygous for MSH2, MLH1 or PMS2 loss of function mutations [30].

The loss of MMR genes directly or indirectly alters signal pathways, loss of strictly regulated expression of cytokine receptors, transcription factors and cell cycle regulation [5]. Unlike normal stem cells the presence of damaged DNA does not activate p53 induced G1 cell cycle arrest and also the repair helicases XPB and XPD fail to functionally interact with p53 [31,32]. This contributes to the loss of stem cell phenotype, a proliferative advantage and cancer stem cell formation.

The mitotic homologous recombination can result in chromosomal translocations, deletions or inversions with deleterious consequences in the adult or during development. Loss of MMR adds up an additional level of genetic instability in a form of chromosomal rearrangements commonly observed in hematological malignancies. High incidence of lymphoma is reported in Msh2 deficient mouse embryonic stem cells [33].

The Msh2 low and deficient mouse ES cells showed poor or complete elimination of repair function and demonstrated the resistance against the toxic effects of an ethylating agent, N-ethyl-N-nitrosourea (ENU). The ENU-induced mutagenicity under the fully or partially deficient conditions accelerated the lymphomagenesis [34]. Toft et al., showed the increase in mutation frequency at Dlb-1 locus due to loss of Msh2 status in normal intestinal cells predispose to malignancy. The increased colonogenicity was observed in Msh2^-/- ^mouse ES cells after exposure to temozolomide but not cisplatin and contributed to failed repair mechanism and apoptosis in a p53 dependent manner [35].

In a study by Fink et al., Msh2^-/- ^mouse embryonic stem cells showed two-fold resistance to the cytotoxic effects of cisplatin as compared to wild type cells [36]. On the contrary, Claij & Riele found no relation between the MMR capacity of cells and their response to cisplatin in a colonogenic assay in mouse ES cells [37]. However, certain specific changes like mutated p53 [38], increased recombinational repair or increased replicative bypass are collaborated with MMR deficiency in affecting the cytotoxicity of cisplatin.

### Therapeutic implications of mismatch repair in cancer stem cells

In most of the malignancies CSCs constitute less than 1% of total cell population. They are in quiescent state and most of the conventional chemotherapeutic drug therapies are effective in actively dividing cells, so for complete eradication of CSCs, the therapeutic agents either should kill cells independently of the cell cycle or selectively induce cycling of cancer cells. Moreover, therapy should target pathways uniquely used by CSCs. CSCs express various drug transporter proteins which efflux the cytotoxic drugs and protect them from damage. The inhibitors to these transporters make the CSCs sensitive to chemotherapy. Since normal stem cells also express some of these transporters so non selective inhibition of these multidrug resistant genes also kill normal stem cells. The characterization of stem cells markers would help in identification of CSCs from normal stem cells have important implications in elimination of CSCs by specifically targeting the tumor cells [39]. Table 2 lists the human cell surface markers in normal as well as CSCs, which are used to distinguish the two populations of cells.

**Table 2:** Markers expressed in normal and cancer stem cells in humans

***Organ***	***Cancer type***	***Normal stem cell markers***	***Cancer stem cell markers***	***References***
Hematopoietic	Leukemia	CD34^+^CD38^-^Thy1^-^Lin^-^	CD34^+^CD38^- ^Thy1^-^Lin^-^	[47, 48]
Breast	Mammary cancer	CD24^med^	CD44^+^CD24^-/low^ESA^+^Lin^-^	[8, 11]
Brain	Brain tumor	CD133^+^Lin^-^	CD133^+ ^Nestin	[49, 50]
Skin	Melanoma cancer	CD20^-^CD166^- ^Nestin^-^	CD20^+ ^CD166^+ ^Nestin+	[51, 52]
Prostate	Prostate cancer	CD133^+^α_2_β_1_^hi^	CD44^+^α_2_β_1_^hi^CD133^+^	[53]
Tongue, Larynx, Throat and Sinus	Head and neck squamous cell carcinoma (HNSCC)	CD44^-^	CD44^+^	[54]
Pancreas	Pancreatic cancer	CD24^-^CD44^-^ESA^-^	CD24^+ ^CD44^+^ESA^+^	[48]

The wild type MMR expression blocks the homologous recombination between the diverged DNA sequences throughout the genome and thus prevent the stem cell to accumulate further genetic instability. Hematological malignancies and lymphoma arise due to chromosomal rearrangements because of loss of MMR proteins [33]. The efficient mismatch repair mechanisms engage the stem cells to undergo apoptosis when DNA is damaged. For example, hematopoietic stem cells show premature senescence (cellular aging) when they are exposed to ionizing radiation or busulfan [40]. In response to alkylation or oxidative DNA damage, the DNA MMR machinery directly or indirectly stimulates signal transduction, transcription factors, loss of cell cycle regulation that derive cell fate decision pathways including apoptosis. The cells with damaged DNA bases undergo G2 arrest in presence of hMLH1 as well as the checkpoint kinases ATR and Chk1 [41]. The stress kinase p38 is activated in presence of hMLH1 and its inhibition cause cells to bypass G2 arrest [42]. Cisplatin treated cells redistribute hPMS2 to the nucleus and its functional interaction with p73, a p53 related protein increases cell death [43]. Functional hMSH2 and hMLH1 also activates c-Abl, one of the proteins of p73 dependent apoptosis pathway [44,45]. The MMR proteins allow the cisplatin-induced initiation of G1 arrest by cyclin D1 degradation [46]. Defective MMR along with the mutated p53 response contributes to cisplatin resistance [38].

**Figure 2:**
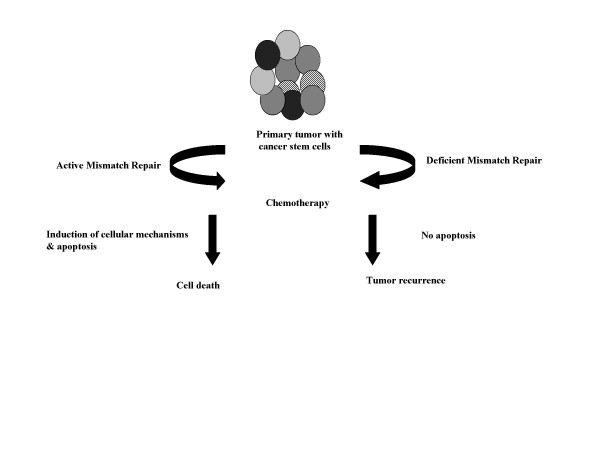
Active mismatch repair system in primary tumors help in the induction of cell death after chemotherapy. Primary tumors containing heterogeneous population of tumor cells along with small % of cancer stem cells (CSCs, represented by dark gray colored oval shape undergo chemotherapy. Presence of active mismatch repair induces cellular response followed by apoptosis, which lead to cell death. However, mismatch repair deficiency makes tumor cells insensitive to drug and it relapses.

Most of the human cancers are identified having mismatch repair deficiency that result in defect in cell cycle and altered growth as in case of hematological malignancies. The defective MMR status in a damaged cell directly contributes to the resistant phenotype against the cytotoxic effects of chemotherapeutic drugs and thus these CSCs survive chemotherapy (Fig 2). The activation of DNA MMR system would help in inhibiting the survival mechanisms by raising the sensitivity of such cells to therapeutic drugs and reduce its tumor potential by arresting their growth. Owning to mass effect, progeny cancer cells still show the symptoms in the patients and therefore, the combinational therapy for the eradication of cancer stem cells and differentiated cells would be more effective. Antitumor treatment strategies selectively targeting the subset of tumor stem cells would be of clinical significance.

## Conclusion

The maintenance of genomic stability is the prime requirement for the stem cell phenotype and its normal functioning. The increased mutation rate and absence of MMR may give rise to stem cell failure, a proliferative advantage and cancer stem cells formation. The cancer stem cells having MMR deficiencies make them insensitive to the treatments against the cytotoxic agents and would increase the risk of relapse and metastasis. The importance of MMR in designing the therapeutic strategies specifically targeting the tumor cells is being explored and pursued in the clinical trials.

## Competing interests

The author(s) declare that they have no competing interests.

## Authors' contributions

The manuscript was written and finalized by the author.
